# Acoustic Field Enabled Polymeric Nanoparticle Deposition onto Vessel Walls for Enhanced Drug Delivery

**DOI:** 10.1021/acs.nanolett.5c01438

**Published:** 2025-05-23

**Authors:** Jianlei Wu, Liang Zhao, Evan H. Dubrunfaut, Valerie A. Lallo, Siyu Chen, Qianhong Wu, Bo Li, Laura G. Bracaglia

**Affiliations:** † Department of Chemical and Biological Engineering, 8210 Villanova University, Villanova, Pennsylvania 19085, United States; ‡ Hybrid Nano-Architectures and Advanced Manufacturing Laboratory, Department of Mechanical Engineering, Villanova University, Villanova, Pennsylvania 19085, United States; § Cellular Biomechanics and Sports Science Laboratory, Department of Mechanical Engineering, Villanova University, Villanova, Pennsylvania 19085, United States

**Keywords:** acoustic field, polymeric nanoparticle, drug delivery, tissue engineering

## Abstract

Polymeric nanoparticles (NPs) are promising tools for transporting and localizing therapeutics with intravenous delivery. Targeting these vehicles to specific tissue sites is challenging. Here, we investigate the use of low-frequency acoustic fields to drive polymeric NPs from circulating blood onto blood vessel walls by using concepts of elastic material deformation. By varying the shear flow rate and duration of acoustic field exposure, we achieved a 1000-fold increase in NP fluorescence intensity on vascular tissue compared with no acoustic field at a flow rate of 2 m/min. Interestingly, we found that acoustic-field-enhanced NP deposition is independent of NP surface chemistry. We also showcase a 100-fold increase in the area of fluorescence detected following NP delivery to an intact, *ex vivo* human vessel wall when a localized acoustic field is applied. This work suggests that local administration of acoustic fields can control polymeric NP biodistribution after intravenous delivery and enhance the treatment of tissue-specific pathologies.

## Graphical abstract

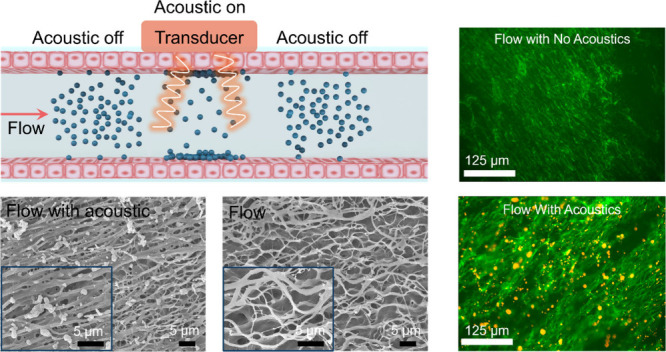

Polymeric nanoparticles (NPs) used for drug delivery are advantageous in comparison to other vectors such as lipids or biological macromolecules due to their material stability; they can protect cargo from degradation in circulation or by metabolism, which can ensure that the active pharmaceutical ingredient remains effective over an extended period. Additionally, polymeric drug delivery vehicles can offer controlled and sustained release of therapeutics, thereby reducing the frequency of medical administration to improve patient compliance.^1^ Due to these strengths and tunable attributes, polymeric drug delivery vehicles are in rapid development and have shown great potential in applications such as cancer therapy,^2–4^ vaccinations,^5^ gene therapy,^6–8^ antimicrobial therapy,^9–11^ cardiovascular disease therapy,^12^ and theranostics.^13^

Therapeutic outcomes in these applications would be enhanced with more controlled biodistribution and reduced off-target accumulation of vehicles delivered intravenously. There are a few factors that can be manipulated to control this. Most simply, biodistribution of polymeric NPs can be influenced by their size^14–16^ and surface chemistry. For example, pegylation of polymeric NPs reduces NP aggregation and can prolong circulation time in the body.^16,17^ Alternatively, targeting ligands can be grafted onto polymeric NPs to direct NP binding to cellular receptors which may be tissue- or disease-specific, improving control over biodistribution at a cellular level.^18,19^ This strategy may not be effective for every purpose since it requires a unique and abundant cell surface molecule to produce specific targeting effects. These strategies are still plagued with unwanted NP accumulation in healthy tissues due to clearance by the RES system or nonspecific binding,^18,20^ reducing the effective dose of NPs.

A strategy that localizes NPs after intravenous administration without relying on biological markers and molecular targeting would be useful. Here, we propose using ultrasound to drive polymer NPs from circulating blood onto endothelial cells lining the vessel wall in a localized manner. From our previous work, we found that low-frequency (i.e., 40 kHz) acoustic fields increase the kinetic energy of NPs (e.g., carbon black and SiO_2_) in water and drive NPs to collide with soft polydimethylsiloxane (PDMS).^26–28^ The viscoelastic PDMS experiences a deformation–recovery process after a collision, capable of dissipating the kinetic energy of NPs, thereby resulting in NP deposition. Inspired by this phenomenon, we propose using a localized acoustic field to drive polymeric NP deposition from circulating blood onto targeted vessel walls. Low-frequency acoustic fields (20–100 kHz), compared to therapeutic ultrasound (1–3 MHz), are particularly effective for NP movement^21^ due to an induced higher cavitation and violent bubble collapse in solution, which generates shock waves and microjets that can kinetically energize NPs.^22^ Low-frequency acoustic fields are observed to be strong enough to drive diverse NPs, including polymers, metal oxides, and even heavy metals.^23–25^ Importantly, more localized particle delivery can be achieved with low frequencies than with homogeneous transport at higher frequencies.^22^ It should be noted that as a physically noninvasive technique, acoustic technology in conjunction with biomaterials has been safely used in biomedical and clinical treatments.^29,30^ Our work, however, deviates from the typical applications in which an acoustic field is used either to stimulate a spatiotemporal drug release from NPs by destabilizing the membrane or shell of NPs or to increase permeability in barrier cells (i.e., sonoporation and sonopermeation) to improve transport.^31,32^

A schematic of low-frequency acoustic-field-induced polymeric NP deposition onto tissues from blood circulation is shown in Figure 1a. We hypothesized that the acoustic field induces polymeric NP deposition more than circulation or flow without an acoustic field. As a demonstration, we utilized a section of bovine aorta (histology image, Figure S1a) as the target tissue source and continuously dipped it inside an aqueous suspension of fluorescently labeled cationic NPs with an average speed of 2.0 m/min to simulate blood flow through the blood vessel. The cationic polymer NPs were formulated from poly­(amine-*co*-ester), a specialized polymer formulation for nucleic acid transport into cells.^16,33^ The dipping setup is shown in Figure S2. It should be noted that this dipping process^34^ is different from the conventional dip-coating process (i.e., dip in and out of solution). The bovine aorta was always immersed in the solution, thereby excluding evaporation-driven deposition.^28^ During the dipping process, an acoustic field (40 kHz, 60 W) was applied to the solution. After 60 s, we observed uniform NP deposition on the endothelial side of the bovine aorta (Figures 1b and 1c, left images). Conversely, only trace amounts of NPs were deposited onto the tissue without an acoustic field (Figures 1b and 1c, right images). At least five images were taken from multiple locations of each sample to ensure data repeatability. Full surface coverage of the tissue by NPs was not achieved with the acoustic field, as compared to previously studied soft polymers, due to the heterogeneity of tissue surface.^35^

**Figure 1:**
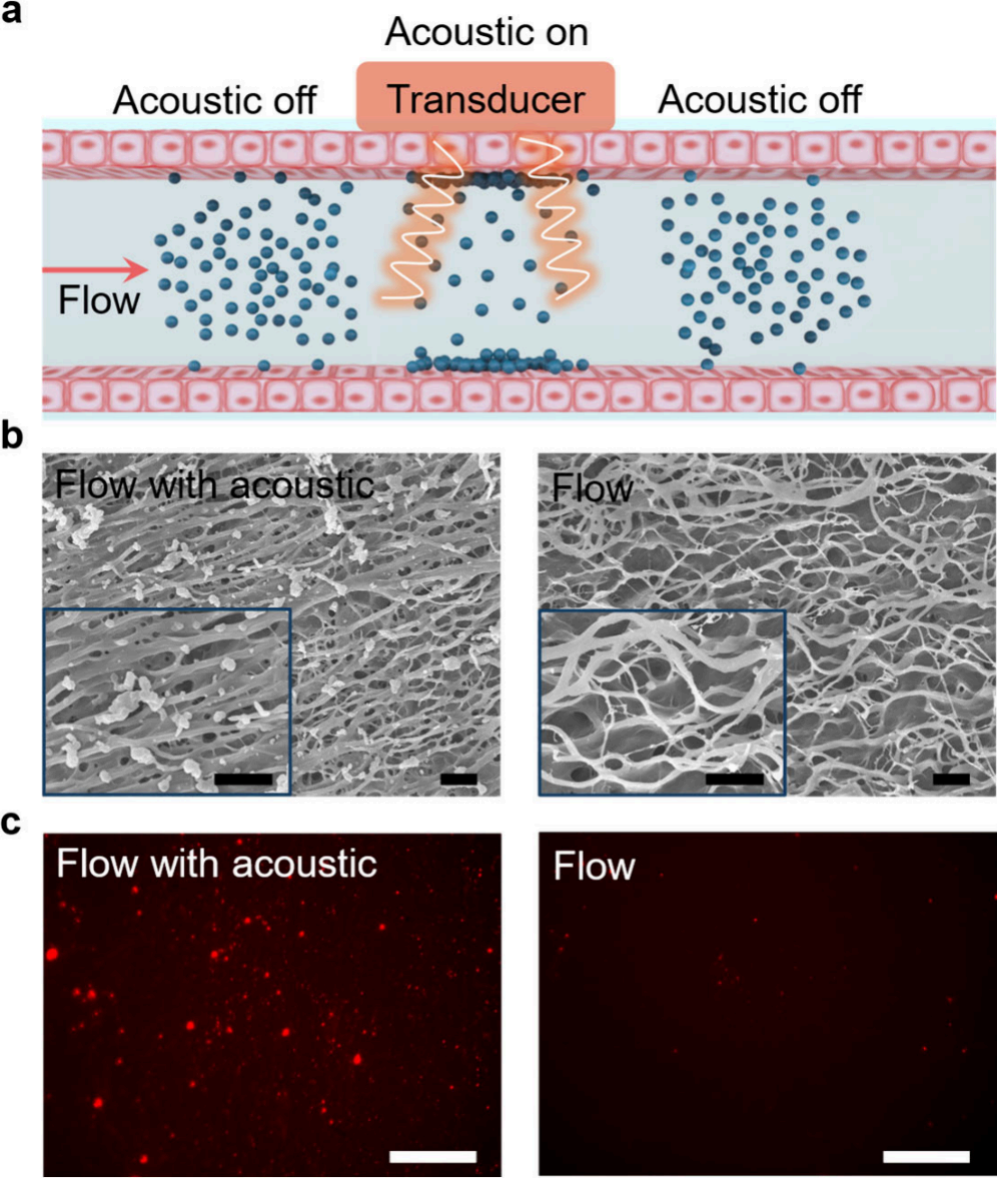
Acoustic-field-assisted NP deposition onto bovine aorta tissue. (a) Schematic of polymeric NP deposition process with acoustic field on and off. In the lumen of a vessel, blue spheres represent the NPs, which are deposited onto the endothelial wall of the vessel. (b) Representative SEM images of cationic NP deposition with acoustic on and off (five images from each of three samples). Scale bars, 5 μm. (c) Representative fluorescent images of tissue *en face*, showing cationic NP deposition with acoustic on and off (five images from each of five samples). NPs are visualized using an encapsulated red fluorescent dye. Scale bars, 100 μm.

Considering that flow rates in body circulation vary by vessel diameter, we investigated the impact of the duration of acoustic exposure combined with different flow rates on cationic NP deposition onto the surface of bovine aorta tissue. NP deposition was observed and quantified through fluorescent images after varied flow rates (representing dipping speeds) and duration times of acoustic exposure (Figures 2b and 2c). Area of fluorescence detected as well as total NP fluorescence intensity above background fluorescence (schematic of data processing shown in Figure 2a, right) were used to compare NP deposition between groups. In these fluorescent images, black regions represent the aorta surface, while white spots indicate cationic NP locations. Both fluorescent imaging and quantification of NP deposition reveal that cationic NPs barely deposit onto the bovine aorta surface in a static deposition (i.e., only acoustic exposure). Furthermore, cationic NPs can be deposited in small amounts onto the bovine aorta when there is no acoustic field applied (i.e., only flow). When the flow rate is constant, more cationic NPs deposit with an increasing duration of acoustic exposure. When the duration of acoustic exposure is held constant, more cationic NPs deposit with an increase in flow rate from 1.1 to 2.0 m/min. When the flow rate further increases to 2.5 m/min, the total NP deposition is reduced. This observation indicates that higher flow rates may introduce too much shear force, making the NPs favor lateral transport. However, some lateral movement of particles is necessary to replenish the NP concentration in close proximity to the vessel walls such that a collision is likely. Overall, a flow rate of 2.0 m/min with 60 s of acoustic duration shows the highest NP fluorescence intensity on the surface of bovine aorta, which means maximum NP deposition. While acoustic field exposure alone limited NP deposition, it significantly enhanced NP deposition upon combination with an appropriate flow rate. We attribute this enhancement of NP deposition to increased collision probability between nanomaterials and the polymer substrate, and colliding with more energy to deform the membrane and result in deposition.

**Figure 2:**
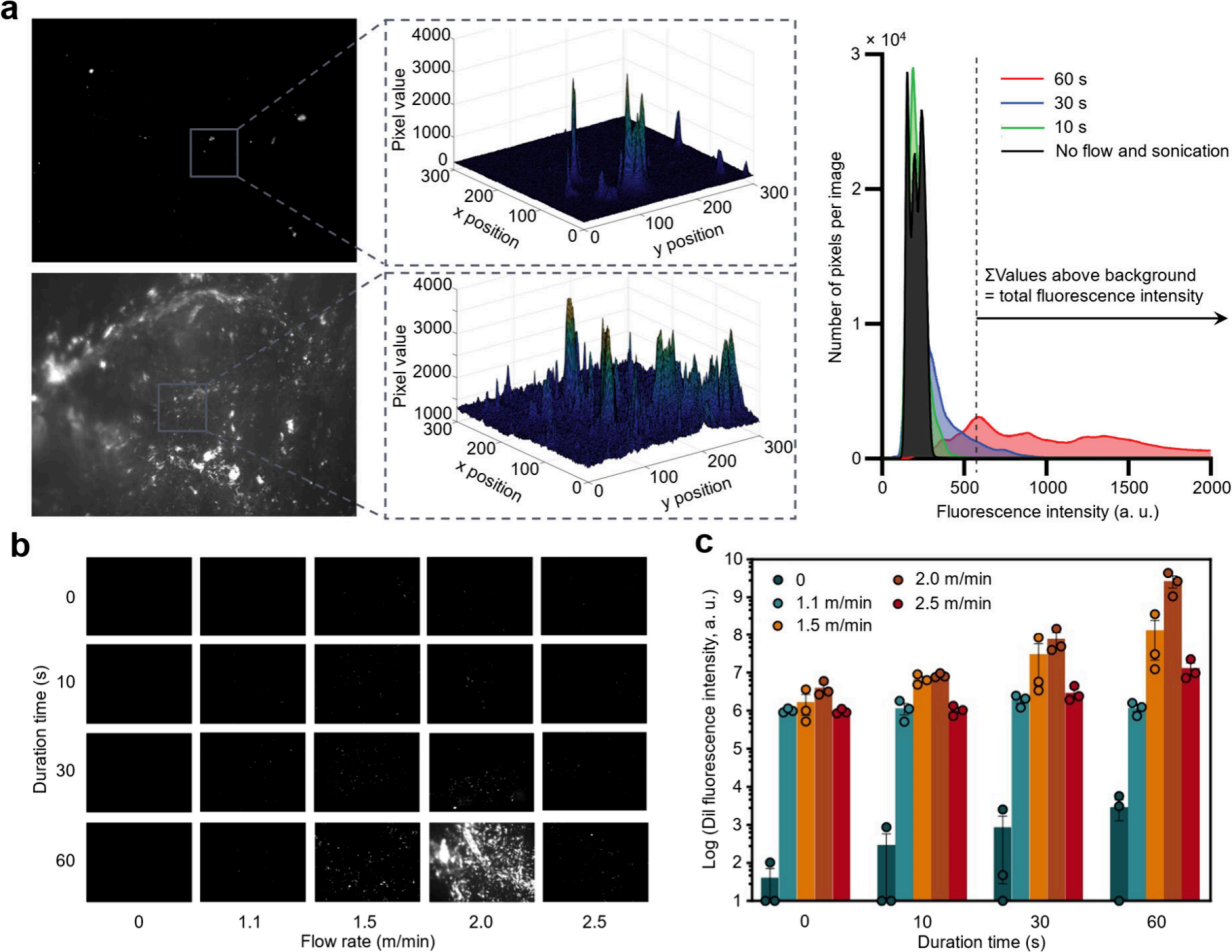
Quantitative examination of acoustic-enabled NP deposition on bovine aorta tissues. (a) Fluorescent images (left) of cationic NP deposition on bovine aorta for 10 and 60 s of acoustic field exposure with an identical flow rate of 2 m/min and corresponding 3D representations (middle) of fluorescent pixel values from deposited NPs within a 300 μm × 300 μm area and histogram of fluorescent intensity (right) of NP deposition under different acoustic exposures. (b) Representative fluorescent images with different acoustic duration times and flow rates. Due to wide range of fluorescence intensities captured, the display settings are varied by flow rate. For a flow rate of 0 m/min, pixels are compressed from a maximum value of 4095 to 1000. For a flow rate of 2.0 m/min, pixels are compressed to 3000. For flow rates at 1.1, 1.5, and 2.5 m/min, pixels are compressed to 1500. (c) Fluorescence intensity of deposited NPs on bovine aorta with different flow rates and acoustic duration times. Each data point is an average of 10 fluorescence images and each condition was completed for an *n* = 3. The two-way ANOVA test was done for the effect of acoustic duration time and flow rate on NP deposition (NP fluorescence intensity). The *P* value of acoustic duration time is below 0.0001 and DF = 3. The *P* value of flow rate is below 0.0001 and DF = 4. The test demonstrates that both acoustic duration time and flow rate show great significance for NP deposition.

As described, NP deposition is quantified using a sum of fluorescence intensity from a fixed area of tissue and the background corrected from tissue without NPs. This is an indirect method to quantify NP deposition and does not necessarily correlate with the physical space consumed by NPs. To relate area of fluorescence with the physical area of NPs, we have also quantified representative SEM images, which display the surface (Figures S3c–S3f) and a cross section of the aortic tissue (Figure S4). This imaging method also shows enhanced NP deposition with the addition of ultrasound.

In addition to cationic NPs, we chose two more polymer NP types, pegylated NPs and poly­(lactic-*co*-glycolic) acid (PLGA) NPs, for acoustic field enabled deposition. As seen in Figures 3a–3c, they all feature a quasispherical shape that is similar to the cationic NPs. However, these NPs have different surface charges and sizes compared to cationic NPs (Figure 3d), which are parameters that may affect the deposition. We have reported in previous work the correlation between NP size and aggregation, which would hinder deposition.^28^ The average hydrodynamic diameters reported are popular sizes found in polymeric drug delivery vehicles, so testing this range for polymeric vehicles is important.

**Figure 3:**
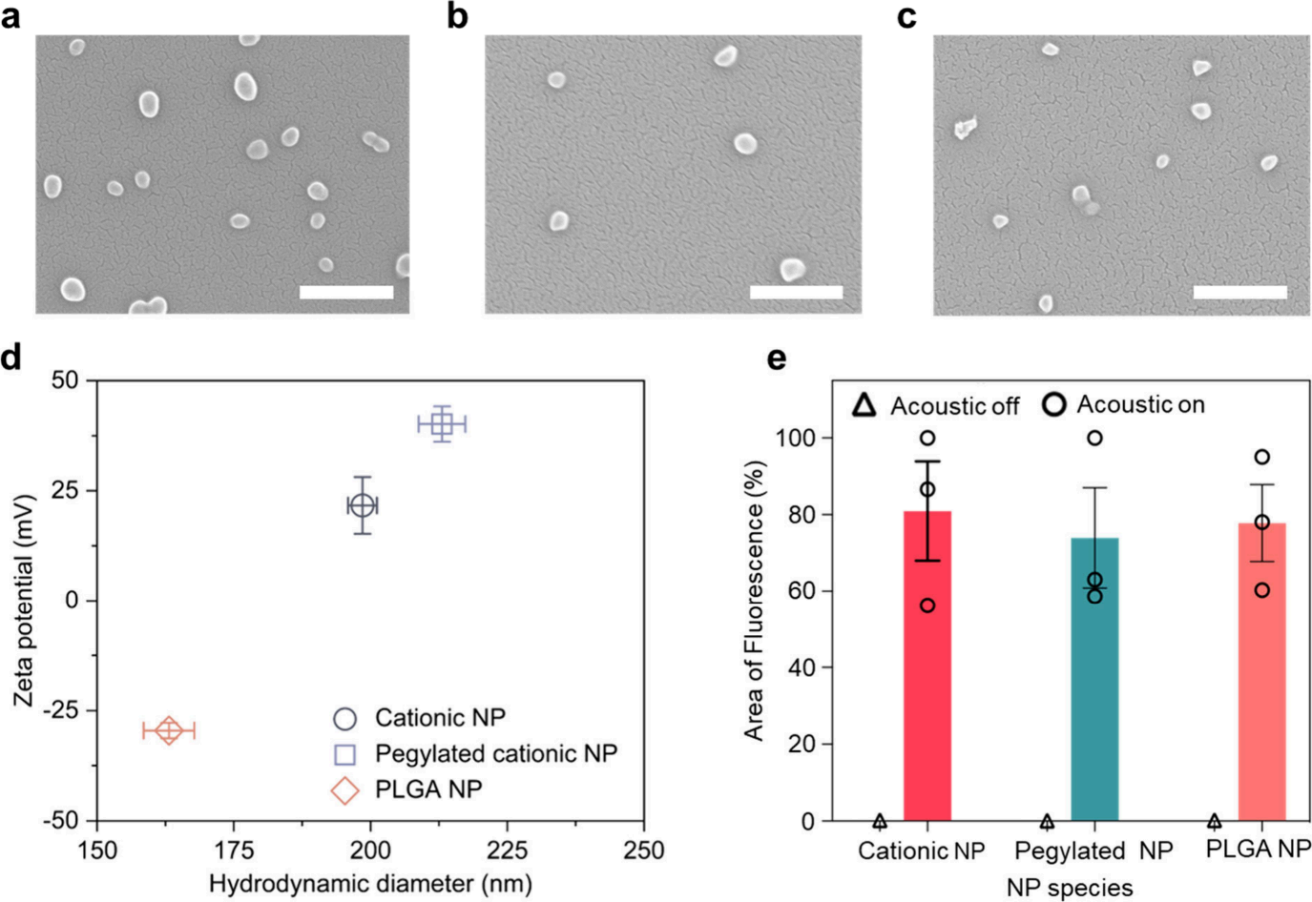
Acoustic field enabled key NP deposition onto bovine aorta. SEM images of (a) cationic NPs, (b) pegylated NPs, and (c) PLGA NPs. Scale bars, 500 nm. (d) Zeta potential and average hydrodynamic diameter for different NPs; error bars represent multiple measures of the mean. All NPs have a PDI of <0.15. (e) Quantification of key NP fluorescence coverage onto bovine aorta tissues. An ANOVA was done to compare NP fluorescence area among the three types of NPs. *P* is 0.9203 (>0.05), which demonstrates NPs with different surface characteristics show no significance to the average NP fluorescence area.

Keeping the same flow rate (i.e., 2.0 m/min) and acoustic field exposure time (i.e., 60 s), which resulted in maximum deposition, we evaluated the NP coverage on bovine aorta for three NP species (Figure 3e). Due to the difference of DiI fluorescence intensity among the three types of NPs, the area of fluorescence from NPs was measured as opposed to total fluorescence intensity. The area of fluorescent signal is nearly zero for each type of NP at a flow rate of 2.0 m/min with acoustic off for 60 s, which indicates minimal NP attachment onto the bovine aorta. With acoustic on for 60 s, the average NP fluorescence area for cationic NPs, pegylated NPs, and PLGA NPs reached 80.93%, 73.91%, and 78.02%, respectively (Figure 3e). Additional SEM images of these samples are shown in Figure S5).

Although the average fluorscence coverage varies slightly for three species of NPs, they are all over 70%. These results demonstrate that acoustic field enabled deposition is not necessarily affected by NP surface charges and size, making this method universally applicable for drug delivery.

To further validate this method for use *in vivo,* an artery isolated from a human umbilical cord was used in an isolated vessel perfusion system to mimic vascular flow within the human body.^36^ Histological images of the artery, obtained from the umbilical cord, show the endothelial surface as well as the tunica media and tunica externa layers intact (Figure S1b), which together give the vessel wall its viscoelastic properties. Cationic, fluorescently labeled NPs were administered in parallel circuits to two isolated vessel segments under 4.0 mL/min flow. This volumetric flow rate was calculated from the linear velocity which led to maximum NP deposition in previous experiments using the tubing diameter of 1.6 mm. One vessel was exposed to the acoustic field for 60 s, and the other was not exposed to acoustics (Figure 4a). Both fluorescence imaging (Figures 4b and 4c) and quantification of NP fluorescent intensity (Figure 4d) show significant NP attachment onto the artery after 60 s acoustic duration with flow at 4.0 mL/min, while NPs barely deposit onto the artery wall under static conditions. This specific flow rate (4 mL/min) is a good approximation of blood flow at times in smaller arteries and capillaries near the external surface of the body (i.e., temporal artery,^37^ finger,^38^ and dorsalis pedis artery^39^).

**Figure 4:**
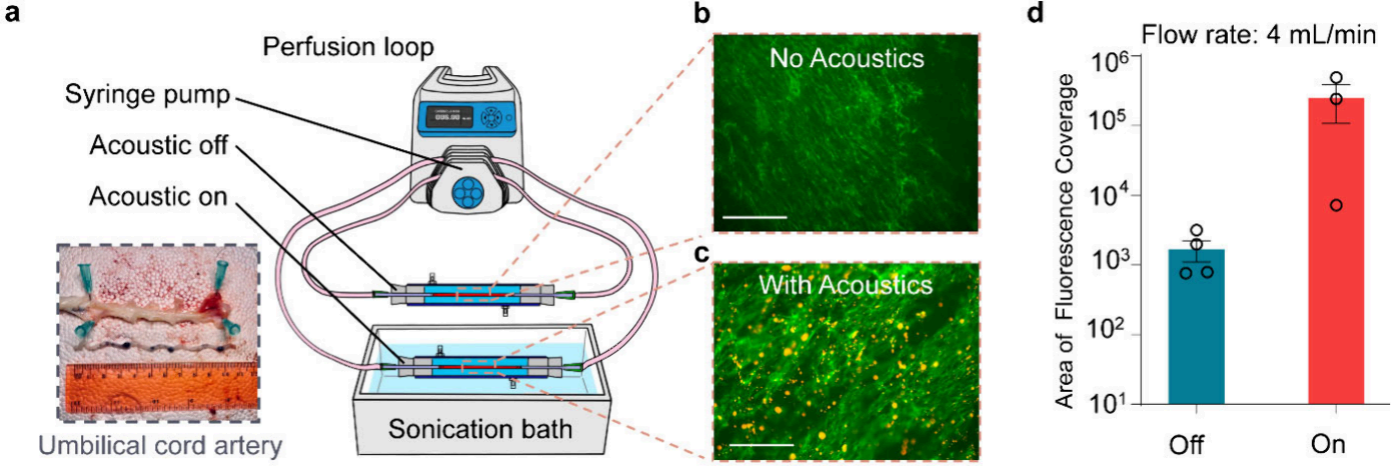
Cationic NP deposition on artery during perfusion. (a) Schematic of 3D flow with acoustics on and off. The inset image is an isolated artery from the umbilical cord. Fluorescent images of cationic NP deposition on artery with acoustic (b) on and (c) off. The artery is stained with fluorescently labeled CD31 antibody to mark the membrane of endothelial cells, and the artery is then cut and imaged *en face*. NPs are displayed in red (DiI dye encapsulated). Scale bars, 125 μm. (d) Quantified fluorescent area of NP deposition with acoustic on and off. Each data point represents an average of 10 fluorescent images, and each condition includes samples from two vessels. A two-tailed unpaired *t* test was done to demonstrate acoustic exposure effect on NP deposition. *P* = 0.09 (<0.1) shows the significance of acoustic exposure for NP deposition onto the artery tissue.

Given this result, we are optimistic that this technique will allow for local control over NP deposition after intravenous administration in a larger and more complex circuit, such as in an animal model. Beyond that, we envision this technique would benefit NP-based drug delivery for the treatment of regionally isolated pathologies, such as diabetic foot ulcers, peripheral nerve damage, and perhaps organ specific tumors. Acoustics can be applied extracorporeally in a specific region for desired accumulation, which would cause NPs to leave the circulation and deposit to the target location. While acoustic fields can help increase NP deposition onto tissue surfaces, it is still crucial to ensure that more NP deposition can enhance drug delivery, which relies on NP endocytosis into the endothelial cells. In our previous works, and shown in Figure S6, we expect that more NPs adhering to the outside of endothelial cells leads to more NPs endocytosed.^36^ Transport of NPs into cells may even be enhanced by membrane destabilization caused by the ultrasound, as is seen in other applications, but destabilization from a low-frequency acoustic field may not persist as long as endocytosis occurs. We have examined cell damage under acoustic field at the testing conditions using both human embryonic kidney (HEK) 293 cells, which are commonly used as a comparable subject for safety studies (shown in Figure S7), and human umbilical vein endothelial cells (HUVECs), which are primary human vascular cells (Figure S8). Looking immediately after acoustic exposure, we see some damaged cell membranes in HEK293 cells, as evidenced by increased annexin V staining (Figure S7D). This damage is reversed after 24 h. In longer studies in HUVECs, we see very limited cell death or membrane instability that can be tied to the ultrasound treatment. Treated HUVECs continue to thrive in culture out to 72 h (Figure S8). Finally, we note that endothelial cells lining the umbilical arteries exposed to ultrasound appear undamaged, with intact cell membranes (visualized with CD31 immunohistochemical stain), few visible gaps in cell–cell junctions, and few interruptions in the monolayer.

In future work, it will be important to address whether the acoustic field can reach deep into tissues in large models of flow and if there is a depth limit to the arteries that can be targeted. We found a threshold flow rate of 2.0 m/min; thus, this technique may be better for small vessels than vessels with a high flow rate. Future testing could investigate tuning acoustic parameters to optimize NP deposition from blood to the vessel wall.

This acoustic method is an effective and straightforward physical technique to drive the deposition of polymer NPs onto a biointerface and can be applied to multiple polymer NP systems. Specific flow rate with acoustic duration time can be utilized for increased deposition of bulk NPs onto targeted vessel walls, which will further improve endothelial cellular uptake of the NP to enhance the efficacy of drug delivery and medical treatment, and causes little safety concern.

## Supplementary Material



## Notes

Details on experimental methods, nanoparticle materials and formulation, nanoparticle characterization, fluorescence microscopy and quantification, and the human vessel perfusion system and additional figures including histology of the materials, a schematic of the acoustic-based dipping system, SEM images of nanoparticles deposited on materials, endocytosis of cationic nanoparticles into endothelial cells, and cell viability under acoustic fields (PDF)
