# Supplementary material for: Acoustic Field Enabled Polymeric Nanoparticle Deposition onto Vessel Walls for Enhanced Drug Delivery
Source: Nano Lett. 2025 May 23;25(23):9270–6. doi: 10.1021/acs.nanolett.5c01438 (PMC12164510; doi:10.1021/acs.nanolett.5c01438)
Supplement: Supplementary file 1 [file nl5c01438_si_001.pdf]

Supporting Information

Acoustic field enabled polymeric nanoparticle deposition onto vessel walls for enhanced drug delivery

Jianlei Wu[1,4], Liang Zhao[2,4], Evan H. Dubrunfaut[1], Valerie A. Lallo[1], Siyu Chen[3], Qianhong Wu[3], Bo Li[2]*, and Laura G. Bracaglia[1]*

[1]Department of Chemical and Biological Engineering, Villanova University, Villanova, PA 19085, USA
[2]Hybrid Nano-Architectures and Advanced Manufacturing Laboratory, Department of Mechanical Engineering, Villanova University, Villanova, PA 19085, USA
[3]Cellular Biomechanics and Sports Science Laboratory, Department of Mechanical Engineering, Villanova University, Villanova, Pennsylvania 19085, United States
[4]Those authors contribute equally to this work.
*Corresponding author. Email: bo.li@villanova.edu; laura.bracaglia@villanova.edu

Materials and Methods:

Materials

The cationic and pegylated polymers, specifically poly(amine-co-ester) with a 60% PDL molar ratio, were synthesized according to previously published methods[1]. PLGA (50:50, 30000 to 60000 MW) was purchased from Sigma Aldrich and used as received. Dichloromethane (DCM) (HPLC grade, +99.9%) was purchased from Sigma Aldrich. Chloroform (HPLC grade, +99.8%) was purchased from Sigma Aldrich. Dimethyl sulfoxide (DMSO) (HPLC grade, +99.8%) was purchased from Sigma Aldrich. Ultra-pure Phosphate Buffered Serum (PBS) (20X) (PH = 7.5) was purchased from VWR and diluted to 1X PBS for use. Poly-vinyl-alcohol (PVA) (13000 to 23000 MW) was purchased from Sigma Aldrich and was dissolved in deionized (DI) water to make 5% PVA solution for use. HUVEC media was made from Medium 199 1X complete serum-free media (Sigma Aldrich) supplemented with 10% premium grade fetal bovine serum (FBS) (Avantor Seradigm), 1.4% L-glutamine (Thermofisher) and 1% penicillin-streptomycin (LifeTech). 5 mg/mL endothelial cell growth supplement (ECGS) reagent was made from Medium 199 1X complete serum-free media, ECGS (VWR) and heparin sodium salt (Sigma Aldrich, 2:1 ratio to ECGS). HUVEC cells were cultured in HUVEC media with an additional 1% ECGS reagent and maintained at 37 °C with controlled humid 5% CO_2 in the incubator. HEK293 cells were cultured in RPMI media (Sigma Aldrich), supplemented with 10% premium grade FBS, 1.4% L-glutamine and 1% penicillin-streptomycin and were incubated at 37 °C with humid 5% CO_2 in the incubator. DiI stain (1,1'-Dioctadecyl-3,3,3',3'-Tetramethylindocarbocyanine Perchlorate ('DiI'; DiIC18(3))) was purchased from Biotium and dissolved in DMSO at 10 mg/mL prior to use. Anti-CD31 PECAM-1 was purchased from Invitrogen. Annexin V FITC was purchased from Invitrogen. Propidium iodide (PI) was purchased from Invitrogen. Antibodies against human CD31 (WM59, MA126196) were purchased from Invitrogen, and detected with the secondary antibody against mouse IgG1 (A32773, Invitrogen). Bovine aorta tissues were purchased from Hung Vuong Food Market in Newark, DE. Optimal cutting temperature (OCT) compound was purchased from Tissue-Tek and used as received. Hematoxylin and eosin Y formula were purchased from Sigma Aldrich and used as received. Bluing reagent was purchased from Abcam and used as received.

NP formulation

PACE NPs (cationic), pegylated PACE NPs and PLGA NPs were formulated from their polymers respectively through an emulsion method. 50 mg of polymer were dissolved into 1 mL DCM in a glass tube (except pegylated PACE was dissolved into 1mL chloroform), and the tube was left overnight to fully dissolve the polymer. 2 mL 5% poly-vinyl-alcohol (PVA) was added into a second glass tube. 15 mL 0.3% PVA was added into a beaker for further NP suspension. DiI dye (0.025 mg) in solution was first added into the DCM tube containing the polymer and the tube was vortexed for 30 s to ensure it was mixed well. The dyed polymer solution was added dropwise to the 5% PVA solution and was then transferred to ice for 10 s. The emulsion in this tube was sonicated for 10 s and left on ice for 10 s three times by a probe sonicator (Cole Parmer Branson SFX250 Sonifier with 1/2" Horn) at 38% amplitude and was then transferred into 0.3% PVA solution to disperse stirring at 400 rpm for 1 min. All the diluted NP solutions were transferred into a round bottom flask and the organic solvent was evaporated via IKA RV8 rotary evaporator at 80 mbar for 15 min. The NP solution was centrifuged twice at 24,000g for 60 mins under 4 °C.

The supernatant was removed, and DI water was added to resuspend the NPs under the ultrasound bath. Finally, the concentration of NP solution was obtained through lyophilization, and NP solution was flash frozen and stored at -80 °C for long term use.

NP characterization

The scanning electron microscope (SEM) images of samples were obtained using a field emission scanning electron microscope (FE-SEM) (Hitachi S-4800 SEM). The samples were all coated with 6 nm gold. Freeze-dried umbilical artery with NP deposition samples and NPs on SiO_2/Si wafers were processed at 10 kV and 10 μA and at 20 kV and 20 μA. The effective diameter of cationic NPs, pegylated NPs and PLGA NPs were tested by Dynamic Light Scattering (DLS) (90Plus Particle Size Analyzer, NanoBrook Omni Brookhaven Instruments). The Zeta potentials of NPs were measured by Zeta Potential Analyzer (NanoBrook Omni Brookhaven Instruments) using Phase Analysis Light Scattering (PALS) mode. All NPs were dispersed in DI water and the concentration was fixed to 0.2 mg/mL in the polystyrene cuvettes. Both DLS and zeta potential tests were measured three times to obtain average effective diameter and average zeta potential. The error bars are derived from the standard deviation of three values.

Human artery tissue collection

Deidentified human umbilical cords were sourced from the Labor and Delivery Unit at Lankenau Institute of Medical Research (LIMR) from consenting donors undergoing planned cesarean section. Placentas and umbilical cords were placed in a sealed container on ice and transported to Villanova University for experimentation. The umbilical cord was severed from the placenta and gently rinsed with cold PBS to remove debris and blood clots from the tissue. Using sterile surgical tools, 8 cm sections of umbilical artery were surgically removed from the cord, rinsed with cold PBS, and set aside for vessel perfusion. 2 cm sections were taken to be used for histology.

NP deposition onto bovine aorta

10 mL of NP solution at 5 mg/mL was added to a 50 mL conical tube was thawed and dispersed homogeneously under the ultrasound. The bovine aorta was thawed, cut into small-size pieces (1 cm length with 1 cm width). Each piece of bovine aorta was stapled and placed tightly onto the surface of PDMS substrate. The inner surface of bovine aorta tissue was exposed to air. The PDMS side was inserted into the groove of a PLA-made 3D printed holder. The 3D printed holder was tied onto the dip coater and the 50 mL conical tube containing NP solution was submerged into the sonication bath. After applying the condition of ultrasound exposure and dipping speed, the PLA holder was unloaded, and DI water was used to rinse the surface of bovine aorta tissue to get rid of the free NPs which were not attached to the surface. Then, vacuum grease was applied to the edges of a glass side, and the tissue sample was placed in the center. A cover clip was attached with grease to keep the bovine aorta tissue as flat as possible. The glass slide was then placed under the fluorescent microscope (EVOS M7000) to image the DiI intensity of NP deposition onto the bovine aorta surface in the RFP channel (Brightness at 0.5, Exposure at 0.1, Gain at 1 under 20X). 10 random tissue spots were imaged and collected from each sample of bovine aorta tissue. Each image was processed using a custom Matlab analysis program to obtain its corresponding fluorescence value to evaluate NP deposition above a predetermined background condition.

NP coverage quantification onto bovine aorta via SEM images
As illustrated in Figure S3, the method workflow proceeds as follows[3]: the original image was first divided into a 3×3 grid to mitigate non-uniform background illumination by working on smaller, locally consistent tiles (a). Each tile was then normalized to grayscale and subjected to initial thresholding via Otsu's method; because our images exhibit multimodal intensity distributions (background, fibers, particles), the threshold was manually adjusted and further refined through interactive erasing and painting to isolate particle-covered regions with high precision (b). All refined tile masks were stitched together to form a full-image mask (c), and the resulting mask was overlaid semi-transparently on the original mosaic to compute and visually convey the overall coverage (d).

Human vessel perfusion system (IVPS)
We have utilized an isolated vessel perfusion system (IVPS) to simulate blood flow through the umbilical artery.[2] Two 8-cm sections of umbilical artery were surgically removed from the umbilical cord and gently flushed with cold PBS to remove any blood clots formed after operation, as seen in Figure 4a. In each end of the vessels, 18-gauge blunt-fill luer-lock needles with soldered notches were inserted, and suture was used to tighten each end of the vessels to the needles. The vessels were inserted into an acrylic chamber, and rubber stoppers were used to plug each end and fix the needles in place. The chamber was then filled with cold PBS and kept on ice until perfusion. Masterflex® L/S 14 Precision Pump Tubing was connected to one end of the chambers via male luer-lock-to-1/16" barb connectors. At the end of the tubing, a luer-lock silicone injection port was connected. While keeping the loop open, HUVEC media was pumped into the system using the Masterflex L/S Peristaltic Pump system. Open-loop media perfusion continued for five minutes to ensure the absence of any leakage in the vessel, and to flush any remaining clots, debris and red blood cells from the vessel. The open end of the tubing was then connected to the needle at the open end of the chamber while perfusion continued. A solution of NPs diluted in HUVEC media was injected upstream of the vessel via injection port and closed-loop perfusion was carried out for one minute. For the vessel undergoing acoustics, the chamber was submerged in the sonication bath for the entire duration. After one minute of perfusion, vessels were removed from each chamber, cut from the needles, sectioned into 5 mm sections, and stained using anti-CD31 PECAM-1 monoclonal antibody, to fluoresce HUVEC cell membranes in the GFP channel. After staining, sections were imaged using a fluorescent microscope (EVOS M7000), and presence of NPs in the RFP channel was quantified.

H&E staining
2 cm umbilical artery sections were suspended in cryo-molds with OCT compound and frozen. 4 μm and 10 μm sections were obtained via cryo-sectioning using a cryostat (Leica CM1950). Sections were then stained using a hematoxylin and eosin staining procedure and fixed with formalin.

Viability Testing
HEK293 and HUVEC cells were cultured as prescribed in tissue culture polystyrene plates until confluent. The entire culture plate was exposed to ultrasound between 10 sec, 30 sec, or 60 sec, as indicated. Cells were either immediately harvested and stained for viability, or allowed to recover

in the incubator for the time indicated. After harvest, cells were stained with propidium iodide and annexin V according to manufacturer instructions and then analyzed on a Guava Easycyte Cytometer. Experimental samples are compared to samples killed with heat (95C for 15 min) as a control for the stains.

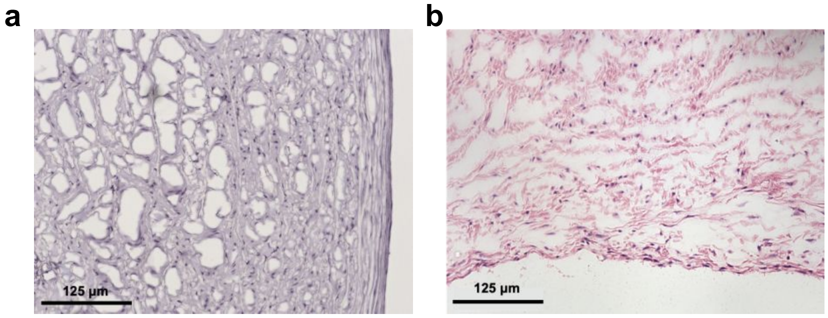

Figure S1. Histology images of (a) plain bovine aorta and (b) human umbilical artery.

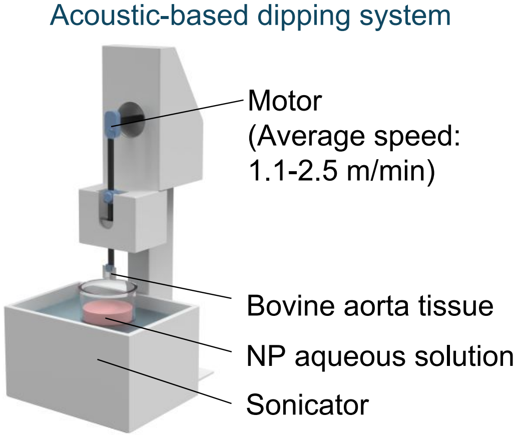

Figure S2. Schematic of acoustic-based dipping system.

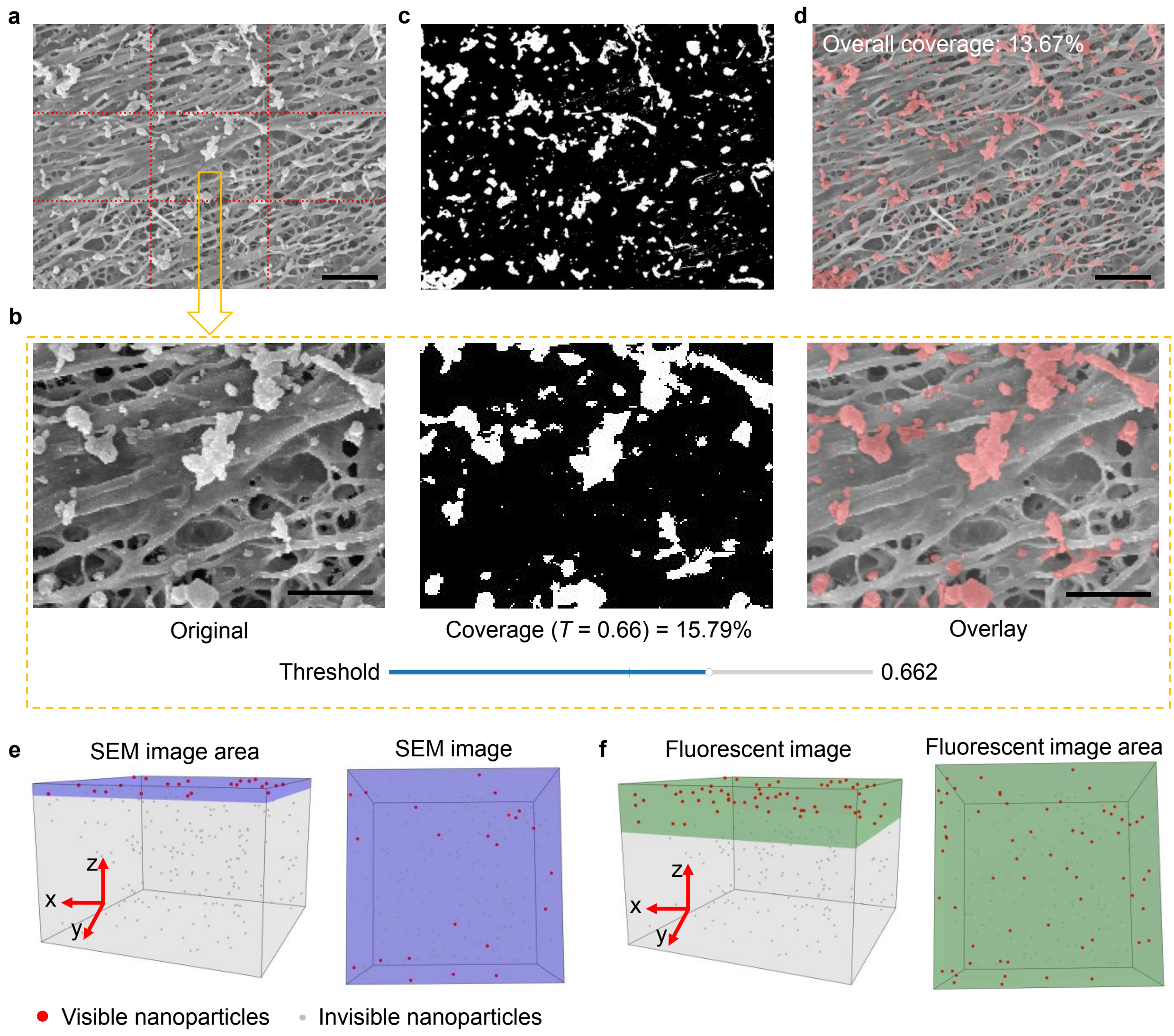

Figure S3. Cationic NP coverage quantification based on SEM images. (a) Original image overlaid with a 3×3 grid. (b) Otsu segmentation on a single tile, followed by threshold adjustment and interactive refinement to produce the final tile mask. (c) Mosaic of all refined tile masks assembled into a full-image mask. (d) Semi-transparent overlay of the final mask on the original image, visually displaying the overall coverage. Scale bars for (a) and (d), 10 μm. Scale bars for (b), 5 μm. Tissue treated with 60 seconds duration of acoustic field and 2.0 m/min flow rate shows 13.67% physical coverage compared to 1.01% of NP coverage on tissue treated with no acoustic field. (e). Schematics on NP deposition coverage quantification using SEM images. (f) Schematics on NP deposition coverage quantification using fluorescent images. Both (e) and (f) methods indicate the difference in NP coverage quantification due to the depth difference that SEM instrument and fluorescent microscope (EVOS M7000) can detect.

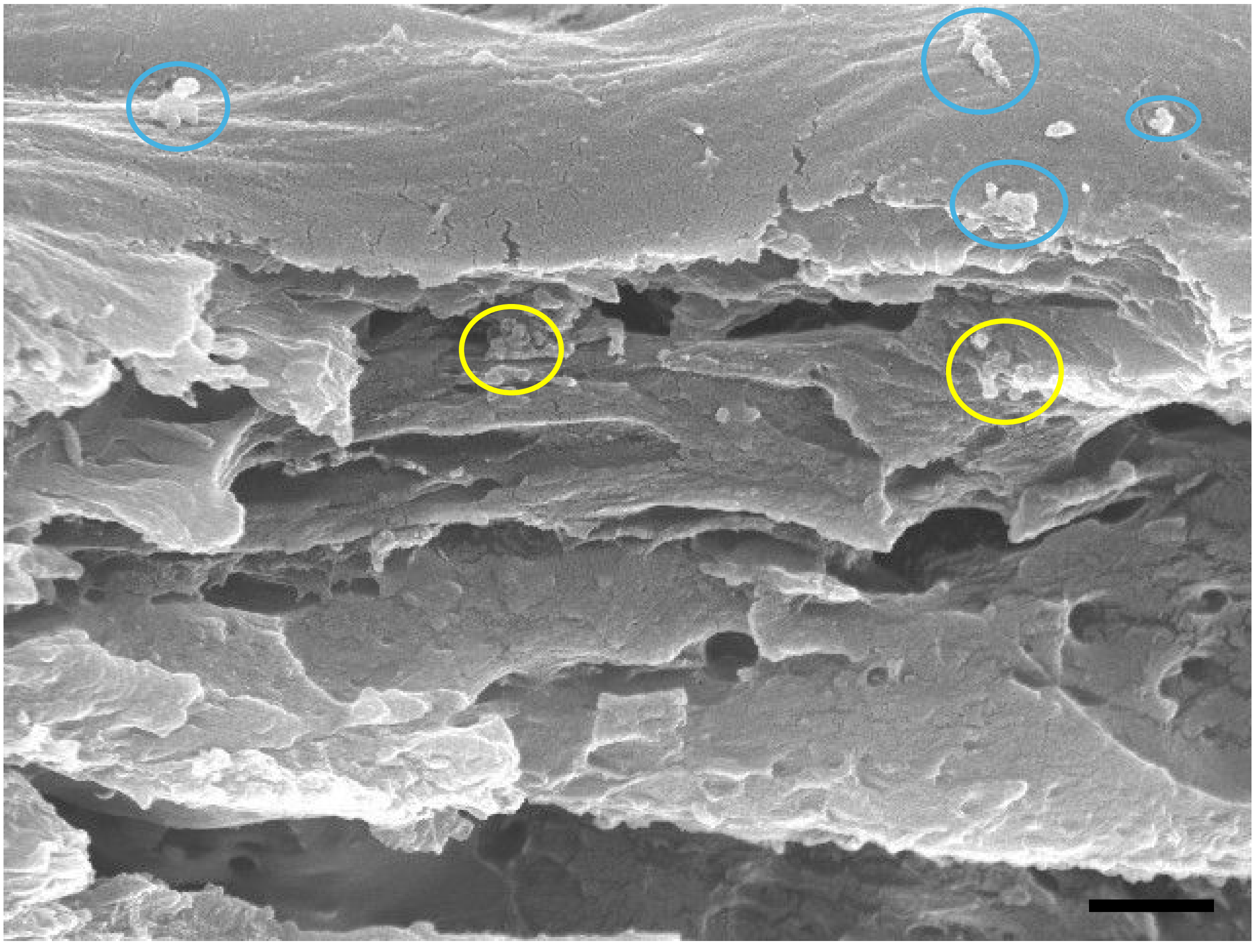

Figure S4. Side view of SEM image of cationic NPs deposited on bovine aorta. The dipping time is 60 s with acoustic on. Blue circles mark the NP deposition on the surface of bovine aorta and yellow circles mark the NP deposition underneath the surface of bovine aorta. Scale bar, 5 μm.

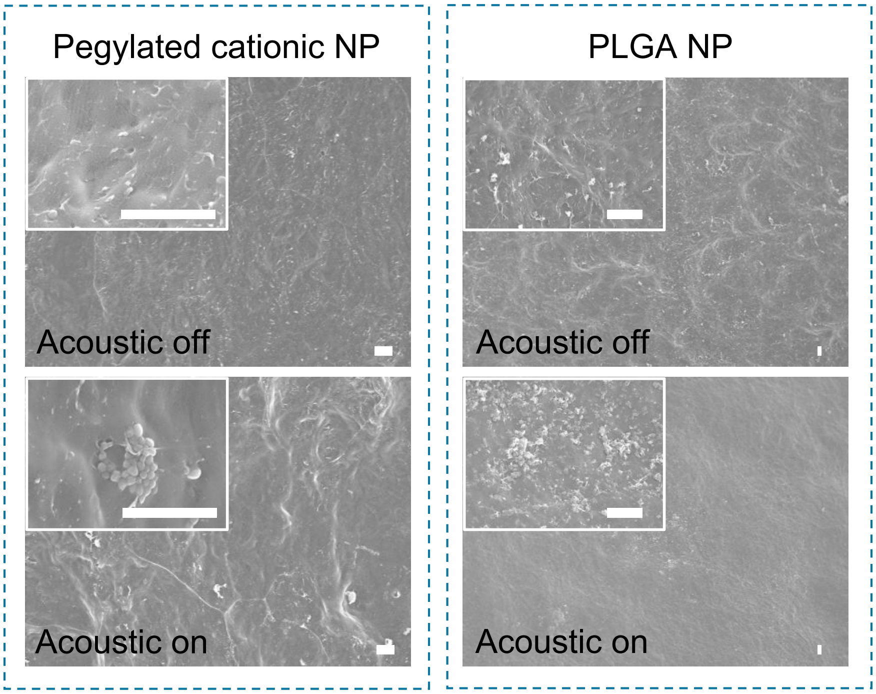

Figure S5. SEM images of different NP deposition on bovine aorta tissues with acoustic on and off. Scale bars, 2 μm.

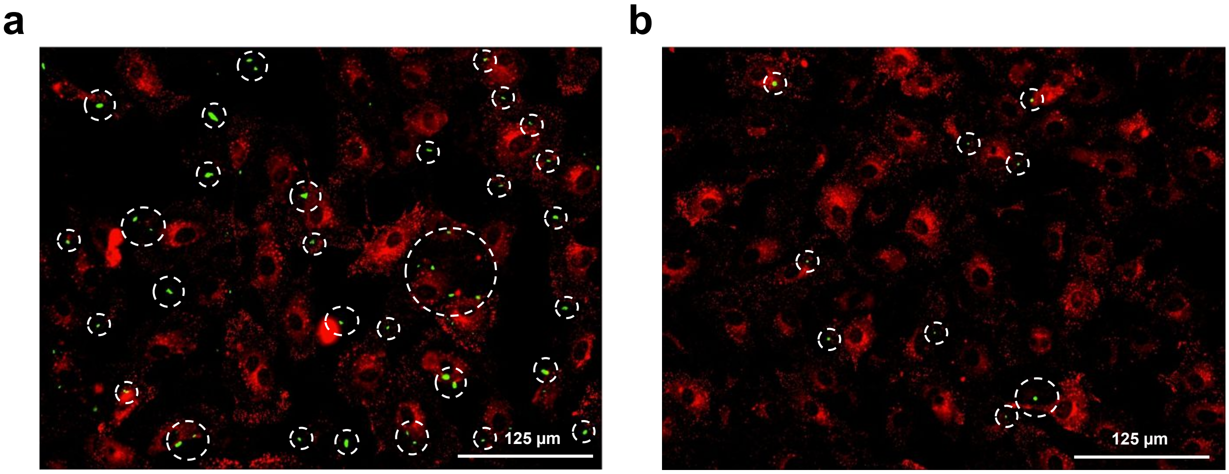

Figure S6. Endocytosis of cationic NPs containing dsDNA after 4 hr at (a) 0.1 mg/mL and (b) 0.05 mg/mL concentration. The red and green represent endosomes and cationic NPs.

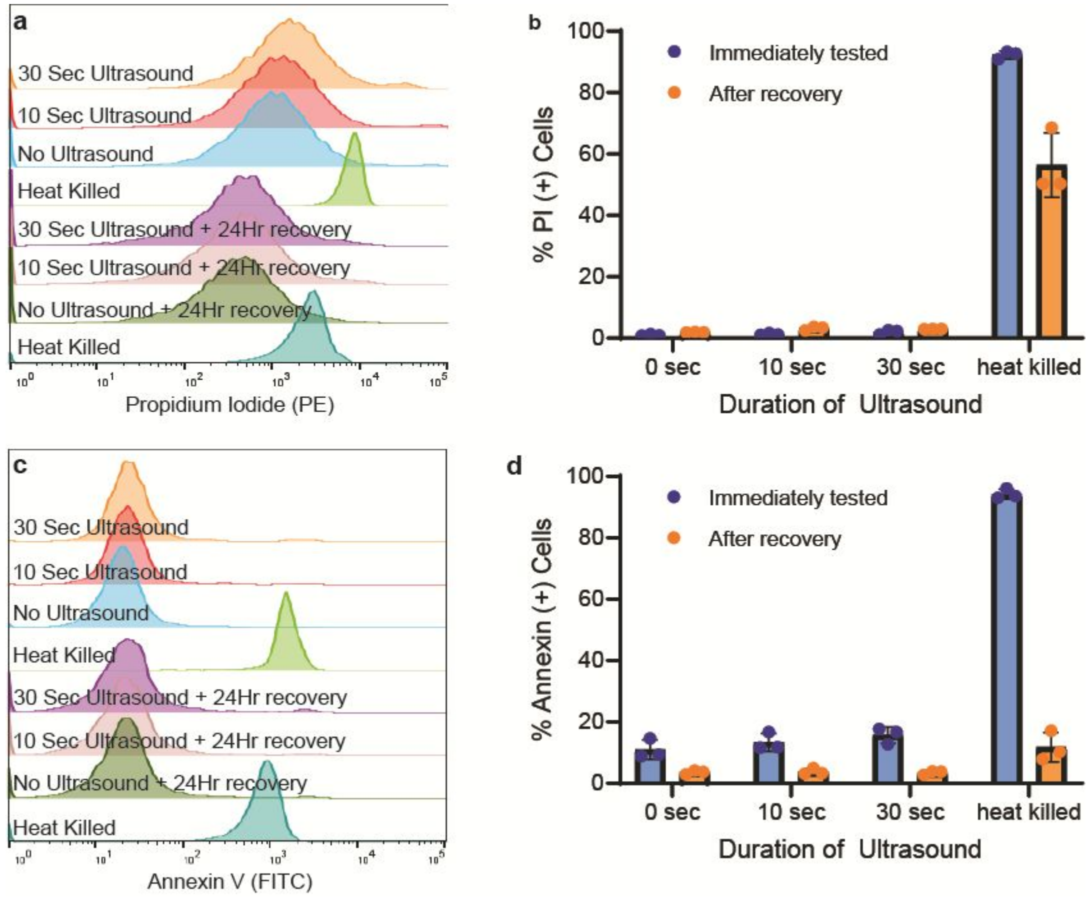

Figure S7. HEK293 cell viability under acoustic field at different time points. (a) Histogram of dead cell population of immediate test and after 24Hr under acoustics of different time points. (b) Quantification of dead cell percentage at immediate test and after 24Hr under acoustics of different time points. (c) Histogram of apoptotic cell population of immediate test and after 24Hr under acoustics of different time points. (d) Quantification of apoptotic cell percentage at immediate test and after 24Hr under acoustics of different time points. All tests include a heat killed cell population as a control. Bars represent the mean of n=3 wells (the mean of each well is indicated with a dot overlaid on the graph).

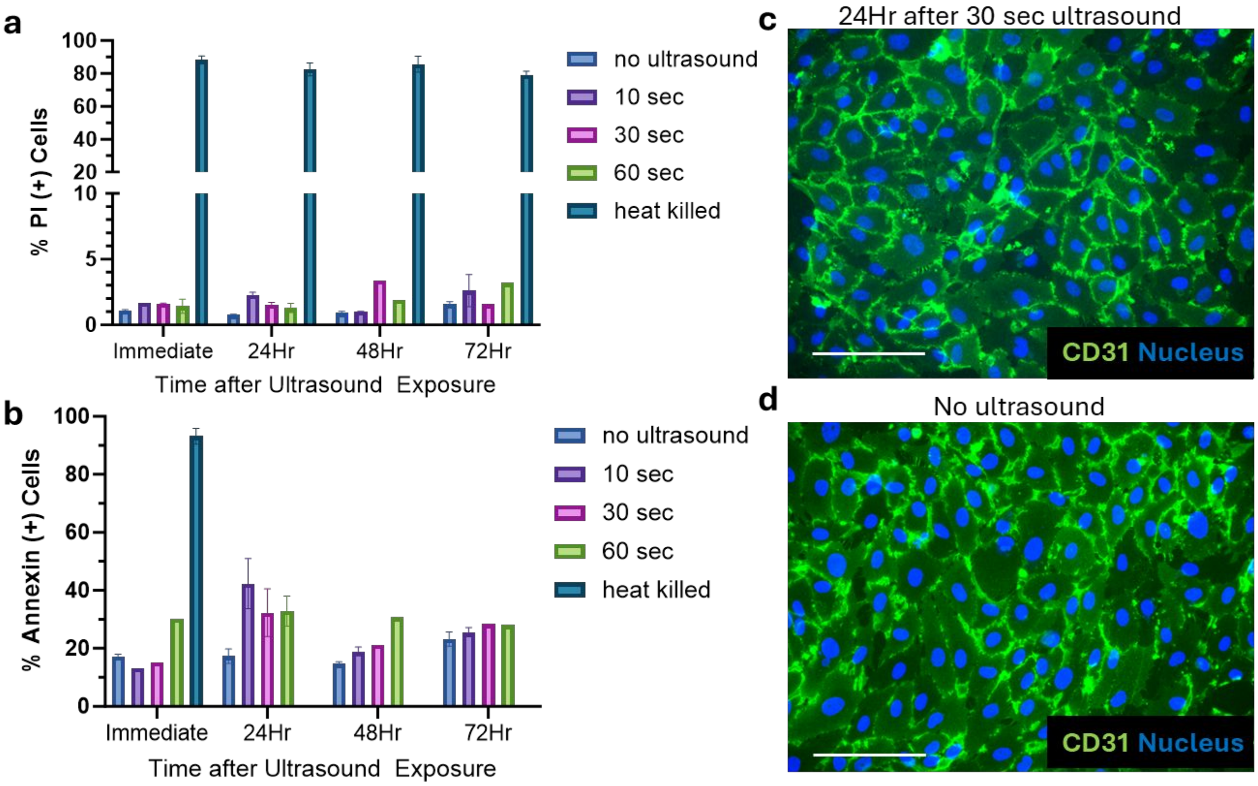

Figure S8. HUVEC viability after acoustic field exposure at different time points. (a) Quantification of dead cell percentage (cells positive for propidium iodide), immediately after acoustic exposure, and after 24Hr, 48Hr, and 72Hr of recovery (n = 3 per test). (b) Quantification of apoptotic cell percentage (cells positive for annexin V), immediately after acoustic exposure, and after 24Hr, 48Hr, and 72Hr of recovery (n = 3 per test). (c) Immunohistochemical staining of HUVECs, 24Hr after ultrasound exposure and without ultrasound exposure (d). CD31, a membrane protein, is shown in green and nuclei are shown in blue. Representative images from 5 images from each of 3 repeats.

References

(1) Kauffman, A. C.; Piotrowski-Daspit, A. S.; Nakazawa, K. H.; Jiang, Y.; Datye, A.; Saltzman, W. M. Tunability of biodegradable poly(amine-co-ester) polymers for customized nucleic acid delivery and other biomedical applications. *Biomacromolecules* **2018**, *19*, 3861-3873.
(2) Lysyy, T.; Bracaglia, L. G.; Qin, L.; Albert, C.; Pober, J. S.; Tellides, G.; Saltzman, W. M.; Tietjen, G. T. Ex vivo isolated human vessel perfusion system for the design and assessment of nanomedicines targeted to the endothelium. *Bioeng. Transl. Med.* **2020**, *5*, e10154.
(3) Sidnawi, B.; Zhao, L.; Li, B.; Wu, Q. A new method for estimating nanoparticle deposition coverage from a set of weak-contrast SEM images. Ultramicroscopy **2024**, 267, 114048.
